# Function, Regulation, and Dysfunction of Intrinsically Disordered Proteins

**DOI:** 10.3390/life11020140

**Published:** 2021-02-12

**Authors:** Giuliana Fusco, Stefano Gianni

**Affiliations:** 1 Centre for Misfolding Diseases, Department of Chemistry, University of Cambridge, Cambridge CB2 1EW, UK; 2 Istituto Pasteur-Fondazione Cenci Bolognetti and Istituto di Biologia e Patologia Molecolari del CNR, Dipartimento di Scienze Biochimiche “A. Rossi Fanelli,” Sapienza Università di Roma, 00185 Rome, Italy

The discovery that a considerable fraction of the eukaryotic proteins lacks a well-defined three-dimensional structure in their native state has revolutionised our general understanding of proteins. In fact, the dogma sequence determines structure determines function constituted, until recently, the foundations of our description of the protein universe. Consequently, it is not surprising that the early years of the intrinsically disordered proteins (IDPs) field have seen some scepticism. In fact, the experimental finding that some proteins did not display a well-ordered structure in isolation was merely considered as an artefact, assuming that the crowded environment of the cell could in fact reshape the native state. Thus, disorder was very far from being considered as a key player in orchestrating some of the molecular events controlling cell biology.

The recent collaborative efforts between experimentalists and theoreticians, however, have contributed in catalysing a revolution in the protein universe and protein disorder has gradually acquired a central role in molecular biology. In this issue of *Life*, a collection of articles is presented with the specific focus of describing the “Functions, Regulation and Dysfunction of Intrinsically Disordered Proteins”. Lermyte et al [1] provided a comprehensive review on IDPs, devoting a particular attention to some disordered systems of relevance for neurological disorders as well as viral infections. The biological properties of IDPs are described vis-à-vis an analysis of the biophysical methods that have been recently optimised to study the structural properties of IDPs. Structural disorder has many faces, and Murciano-Calles [2] review a specific aspect of this by showing how a protein interaction domain class, namely the PDZ domains, have ample and malleable folding landscapes that enable an intrinsic plasticity that enable a connection between diverse binding partners. Protein–protein interactions are crucial in the function and pathology of IDPs. Studying the mechanism of binding by IDPs is a top priority. Visconti et al. [3] used kinetic experiments to characterise the binding between the cancer-related IDP Gab2 and the N-SH2 domain of SHP2, showing how partner recognition occurs in this disordered system.

In the study of IDPs, molecular dynamics simulations (MD) have made fundamental contributions. In this issue, Sullivan and Weinzierl [4] used MD to probe the conformation of the N-terminal 88 amino acids of c-MYC, an oncoprotein that plays a key role in controlling cell proliferation and apoptosis. In the context of IDP interactions, Sala et al. [5] studied the properties of the Sic1 kinase-inhibitor domain (KID) using MD. The results showed that this protein relies on a conformational selection mechanism to recognise the correct molecular partners. Coarse-grained MD simulations were also used by Navarro-Paya et al. [6] to characterise the fundamental binding of α-synuclein (αS), a central IDP whose aggregation is associated with Parkinson’s disease, to synaptic membranes. This protein is completely disordered in the cytosol but undergoes disorder-to-order transition upon binding with biological membranes. New insights on the αS conformations in the cell were provided by Colla and co-workers [7], who developed a set of Forster Resonance Energy Transfer biosensors to distinguish between monomeric and oligomeric conformations of αS in the cellular milieu.

In the context of αS interactions, Burmann and co-workers [8] reviewed the crucial role of molecular chaperones in regulating their physiological functions as well as the pathological aspects. Moreover, the crucial interactions with mitochondria as well as the regulation by posttranslational modifications were elucidated to understand the mechanisms of αS aggregation in the pathological contexts leading to Parkinson’s disease and other synucleinopathies. Other pathological mechanisms involving αS have been proposed. In particular, Pountney and co-workers [9] studied the upregulation of the secretion machinery in the astrocyte response to extracellular αS, suggesting a role in the release of neuroinhibitory and proinflammatory factors in synucleinopathies.

The onset and development of neurodegenerative diseases such as Parkinson’s and Alzheimer’s are inherently associated to the insurgence of oxidative stress in the neurons. The excessive production of free radicals and reactive oxygen species (ROS) have been associated to the mechanisms leading to neuronal death, review by Abramov et al. [10] Intracellular formation of ROS is also originated by metal imbalance. In the context of metal interactions by IDPs associated with neurodegenerative disease, Lucas and co-workers [11] investigated the process of dityrosine crosslinking of αS upon iron binding, with tyrosine 39 resulting as the main contributor to dityrosine and Y125 appearing to be involved in dityrosine crosslinks in unmetalated αS. Similarly important is the interaction of the Abeta IDP with copper(II). In this context, Valensin and coworkers [12] characterised the potent antioxidant rosmarinic acid and its role as a mediator of the copper(II)-induced neurotoxicity. The study showed that rosmarinic acid is able to interfere with the interaction between amyloid β and copper(II) by forming a ternary association.

The main conformational transition of IDPs involved in neurodegenerative diseases involves the formation of fibrillar aggregates with common structural topologies (cross-β spine) and showing peculiar mechanical properties at a microscopic level that make them stronger than steel. Scollo and La Rosa [13] reviewed how the interaction with the membranes is a fundamental aspect of the pathological mechanisms involving amyloid aggregates of otherwise soluble proteins. Visentin et al. [14] studied how the aggregation of neuroserpin in the context of familial encephalopathy can be suppressed in vitro by embelin, suggesting routes of treatments against neuroserpin aggregation. It has also emerged that amyloids are not only pathological accidents but that they can occasionally have a functional relevance. This aspect is discussed in this issue by Rubel and co-workers [15], overviewing the variety of roles that are currently known for functional amyloids.

In summary, we have assembled a collection of articles that study and discuss different aspects of this intriguing class of proteins. More remains to be discovered in the IDP universe, and this elusive state poses the challenges for the next decades to overcome the limitations of current techniques in vitro and in vivo that have mostly been tailored to study structurally defined systems.
